# Anesthetic management of multiple acyl-coenzyme A dehydrogenase deficiency in a series of surgeries under general anesthesia: a case report

**DOI:** 10.1186/s40981-021-00459-3

**Published:** 2021-07-10

**Authors:** Ryoko Owaki-Nakano, Midoriko Higashi, Kohei Iwashita, Kenji Shigematsu, Emiko Toyama, Ken Yamaura

**Affiliations:** 1 Department of Anesthesiology, Fukuoka University Hospital, 7-45-1 Nanakuma, Jonan-ku, Fukuoka, 814-0180 Japan; 2 Department of Anesthesiology and Critical Care Medicine, Graduate School of Medical Sciences, Kyushu University, 3-1-1 Maidashi, Higashi-ku, Fukuoka, 812-8582 Japan

**Keywords:** Acyl-coenzyme A dehydrogenase, Coronary artery bypass surgery, Fatty acid metabolism, Rhabdomyolysis

## Abstract

**Background:**

Glutaric acidemia is a type of multiple acyl-coenzyme A dehydrogenase deficiency, an inborn error in fatty acid metabolism. In patients with glutaric acidemia, during the perioperative period, prolonged fasting, stress, and pain have been identified as risk factors for the induction of metabolic derangement. This report describes the surgical and anesthetic management of a patient with glutaric acidemia.

**Case presentation:**

A 56-year-old male patient with glutaric acidemia type 2 underwent a series of surgeries. During the initial off-pump coronary artery bypass surgery, the patient developed renal failure due to rhabdomyolysis upon receiving glucose at 2 mg/kg/min. However, in the second laparoscopic cholecystectomy, rhabdomyolysis was avoided by administering glucose at 4 mg/kg/min.

**Conclusions:**

To avoid catabolism in patients with glutaric acidemia, appropriate glucose administration is important, depending on the surgical risk.

## Background

Glutaric acidemia type 2 is a type of multiple acyl-coenzyme A dehydrogenase deficiency, which is an autosomal recessive inborn error of fatty acid metabolism. This condition involves promoted protein catabolism and muscle tissue degradation. Metabolic acidosis, hypoglycemia, muscle weakness, and muscle pain are the main symptoms; moreover, there are cases of neonatal deaths or rhabdomyolysis-caused renal failure during adulthood [1]. Its main symptoms in adults include muscle pain and repeated rhabdomyolysis; moreover, it causes life-threatening complications, including acute renal, heart, respiratory, or hepatic failure [2]. Currently, the treatment strategies involve prevention of catabolism through regular feedings, restricted intake of long-chain fatty acids, supplementation with medium-chain triglycerides, and frequent carbohydrate intake in some patients to prevent the activation of fatty acid metabolism [3]. Surgery and anesthesia pose a threat to patients with glutaric acidemia because prolonged fasting, stress, and pain are known risk factors for the induction of metabolic derangement. In patients with glutaric acidemia, special consideration should be given into nutrition management during the perioperative period.

To date, some cases of perioperative management of patients with glutaric acidemia have been reported in the literature [4]. However, to our knowledge, no study has focused on the management of a patient undergoing multiple operations. Herein, we present the case of a patient with glutaric acidemia who underwent a series of surgeries under general anesthesia.

Written informed consent for publication was obtained from the patient. This manuscript adheres to the CARE guidelines.

## Case presentation

A 56-year-old male patient (weight, 82 kg; height, 168 cm) with glutaric acidemia type 2 was scheduled for laparoscopic cholecystectomy for cholecystolithiasis. He had a history of rhabdomyolysis with lumbago and brown urine as initial symptoms at the age of 44 years. At the age of 52 years, he was diagnosed with glutaric acidemia. He did not receive nutrition therapy, including fat restriction; however, he developed rhabdomyolysis approximately once a year and needed fluid therapy. His previous medical history included diabetes mellitus requiring medical treatment, cerebral infarction, and bronchial asthma. Preoperative examination for laparoscopic cholecystectomy led to a diagnosis of angina pectoris with three-vessel disease. Consequently, an off-pump coronary artery bypass grafting (CABG) was planned preceding laparoscopic cholecystectomy.

### Off-pump CABG

**Fig. 1:**
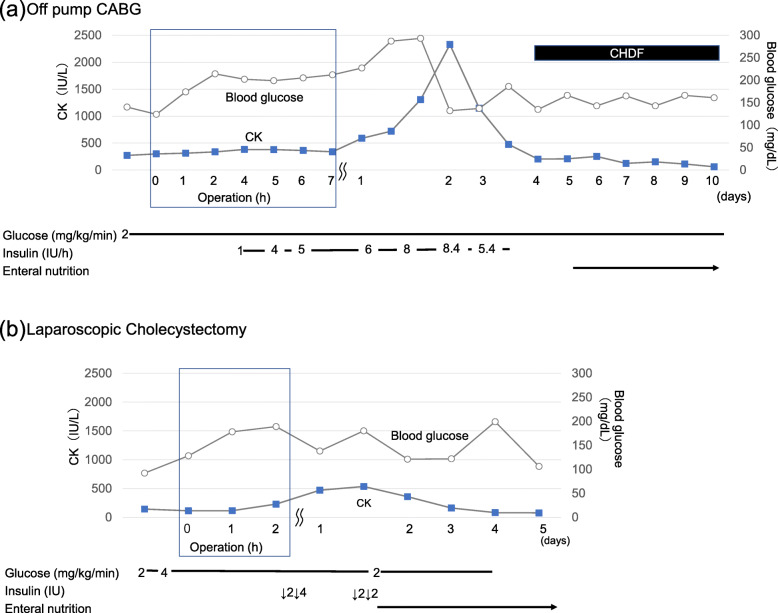
**a** Perioperative blood CK and glucose levels during off-pump CABG. The CK levels increased to a maximum value of 2328 U/L on POD 2. Insulin administration was initiated when the blood glucose levels exceeded 200 mg/dL and continued until POD 3. CHDF was performed between PODs 4 and 25. **b** Perioperative blood CK and glucose levels during laparoscopic cholecystectomy. The CK levels slightly increased on PODs 2 and 3 with a continuous glucose infusion rate of 4 mg/kg/h and intermittent administration of intravenous insulin; however, there was no acute renal failure. CK, creatine kinase; CABG, coronary artery bypass grafting; POD, postoperative day; CHDF, continuous hemodiafiltration

The preoperative serum creatine kinase (CK) and creatinine levels were within the normal range. Metformin hydrochloride, an oral hypoglycemic agent, was discontinued 2 days before the surgery. General anesthesia was induced using remifentanil (0.3 μg/kg/min) and midazolam (6 mg), with rocuronium (70 mg) administered as a muscle relaxant. Anesthesia was maintained using midazolam (0.03 mg/kg/h), dexmedetomidine (0.4 μg/kg/h), and remifentanil (0.1–0.5 μg/kg/min) with O_2_-air mixture. In addition to the standard intraoperative monitoring, direct radial arterial pressure, pulmonary artery pressure, transesophageal echocardiography, and electroencephalogram were monitored. Moreover, the arterial blood pH, glucose, serum CK, serum and urine myoglobin, and lactate levels were measured at 1-h intervals. Administration of 2 mg/kg/min glucose using 10% glucose solution was started from midnight of the day before surgery. It was continued throughout the operation and postoperatively in the intensive care unit (ICU) using 50% glucose solution. Continuous insulin administration was started when intraoperative blood glucose exceeded 200 mg/dL. The operation and anesthesia times were 8 h 2 min and 9 h 49 min, respectively. The myoglobin level started to increase just after surgery. In the ICU, the body temperature was 36.7°C, and postoperative shivering was not observed. Moreover, the serum potassium level was 4.0 mEq/L. The total in-out balance of volume was 2294 ml: intravenously infused fluid (crystalloid and colloids), 4260 mL (2260 mL and 2000 mL, respectively); cell saver autotransfusion, 1600 mL; estimated blood loss, 592 g; cell saver output, 2786 mL; and urine output, 178 mL. The patient was sedated using midazolam and dexmedetomidine; subsequently, postoperative analgesia was maintained through continuous fentanyl infusion. There was an increase in the Mm-CK levels to a maximum value of 2328 U/L, while the serum potassium level increased to 5 mEq/L on postoperative day (POD) 2. Further, the serum myoglobin level increased to a maximum value of 635.8 ng/mL on POD 4. Urine volume decreased to approximately 0.2–0.5 mL/kg/h, and serum creatinine increased to >4.0 mg/dL, which indicated acute renal failure. Consequently, continuous hemodiafiltration was started and continued up to POD 25 (Fig. 1a).

### Laparoscopic cholecystectomy

The patient underwent laparoscopic cholecystectomy 316 days after undergoing CABG. General anesthesia was induced using midazolam (3 mg), remifentanil (0.2 μg/kg/min), and rocuronium (60 mg). Transversus abdominis plane block was performed with an ultrasound linear transducer using 40 mL of 0.25% levobupivacaine after anesthesia induction. Anesthesia was maintained using sevoflurane (1.5%) and remifentanil (0.03–0.15 μg/kg/min). The operation and anesthesia times were 1 h 51 min and 3 h 30 min, respectively. The total infused fluid and urine volumes were 1170 mL and 100 mL (0.36 mL/kg/h), respectively. The glucose infusion rate was 2 mg/kg/min using 10% glucose solution, was started from midnight of the day before surgery, and increased to 4 mg/kg/min using 50% glucose solution when introducing anesthesia. There was a slight increase in the postoperative serum CK and myoglobin levels at a maximum value of 534 U/L and 213 ng/mL on POD 1, respectively. The increased CK and myoglobin levels were promptly normalized. The serum creatinine level was normal, and renal function was not impaired (Fig. 1b).

## Discussion

Glutaric acidemia type 2 is a type of multiple acyl-coenzyme A (CoA) dehydrogenase deficiency. Numerous intramitochondrial pathways for electron transfer are impaired by deficiency of multiple CoA dehydrogenases, such as acyl, glutaryl, and isovaleryl CoA dehydrogenases, thus, leading to decreased energy production from fatty acid. It is characterized by the accumulation of fatty acids in the plasma and urinary excretion of numerous organic acids, including glutaric acid. Glutaric acidemia type 2 has been categorized into the neonatal and late onset types. The late onset type is characterized by repeated episodes of hypoglycemia and proximal myopathy, as observed in our case [5]. Optimal anesthetic management remains unclear [4]. During laparoscopic cholecystectomy, we avoided using propofol; rather, we used sevoflurane. We observed that midazolam, dexmedetomidine, and sevoflurane could be safely used.

It is important to avoid hypoglycemia development in patients with glutaric acidemia type 2 [4], similar to those affected with other fatty acid metabolism disorders [6]. The appropriate glucose amount to be administered is dependent on several factors, including residual enzyme activity, surgery type, and age [6]. For our patient, we administered glucose from preoperative fasting onset until meal resumption to avoid catabolism. A glucose infusion rate of 6 and 8 mg/kg/min is required for healthy children and infants, respectively [7]. In adult patients with other fatty acid metabolism disorders, the recommended glucose infusion rate varies widely with previous reports indicating a glucose infusion rate of 2 mg/kg/min [6, 8, 9]. We employed a glucose infusion rate of 2 mg/kg/min during the perioperative period of CABG. However, the patient developed rhabdomyolysis and acute renal failure. CABG itself also presents a risk of developing rhabdomyolysis of approximately 8–19% [10], while glutaric acidemia is an additional risk factor for the development of rhabdomyolysis. In our case, rhabdomyolysis could have been caused by insufficient glucose infusion rates for the energy requirement during large surgical stress, such as that while undergoing off-pump CABG. Increased CK levels are indicative of catabolism, which causes rhabdomyolysis. This indicates that proper administration of glucose and the use of insulin as needed should be considered by checking the serum CK levels during CABG. In the second surgery, rhabdomyolysis did not occur at a higher dose (4 mg/kg/min) during laparoscopic cholecystectomy. In the case of increased CK levels, additional glucose (6–8 mg/kg/min) with insulin should be administered [6–8, 11]. These findings indicated that the infusion rate of glucose varies depending on the severity of the disease and surgery, and that the glucose infusion rate should be changed according to surgical stress, after checking the serum CK levels.

## Conclusions

We present the case of a patient with glutaric acidemia type 2 who underwent a series of surgeries under general anesthesia. After the initial off-pump CABG, the patient developed renal failure due to rhabdomyolysis, which was attributed to the lower glucose infusion rate. Rhabdomyolysis was avoided in the second surgery through suitable glucose administration while monitoring the serum CK levels. Perioperative infusion rate of glucose should be varied depending on the severity of the disease and surgery, and changed according to surgical stress by monitoring the serum CK levels.

## Data Availability

Not applicable

## Abbreviations

CABG: Coronary artery bypass grafting
CK: Creatine kinase
ICU: Intensive care unit
CHDF: Continuous hemodiafiltration
POD: Postoperative day
